# Protocol of a prospective and multicentre China Teratology Birth Cohort (CTBC): association of maternal drug exposure during pregnancy with adverse pregnancy outcomes

**DOI:** 10.1186/s12884-021-04073-0

**Published:** 2021-09-01

**Authors:** Yangwen Zhou, Jing Tao, Ke Wang, Kui Deng, Yanping Wang, Jianxin Zhao, Chunyi Chen, Tingxuan Wu, Jiayuan Zhou, Jun Zhu, Xiaohong Li

**Affiliations:** 1 National Center for Birth Defect Monitoring of China, West China Second University Hospital, Sichuan University, No. 17 Ren Min Nan Lu, Sichuan Province 610041 Chengdu City, People’s Republic of China; 2 Key Laboratory of Birth Defects And Related Diseases of Women and Children (Sichuan University), Ministry of Education, Chengdu, Sichuan China; 3 National Office for Maternal and Child Health Surveillance of China, West China Second University Hospital, Sichuan University, No. 17 Ren Min Nan Lu, Sichuan Province 610041 Chengdu City, People’s Republic of China

**Keywords:** Drug, Teratology, Birth cohort, Pregnancy, Birth defect

## Abstract

**Background:**

As reported, 27-93 % of pregnant women take at least one drug during pregnancy. However, drug exposure during pregnancy still lacks sufficient foetal safety evidence of human origin. It is urgent to fill the knowledge gap about medication safety during pregnancy for optimization of maternal disease treatment and pregnancy drug consultation.

**Methods and analysis:**

The China Teratology Birth Cohort (CTBC) was established in 2019 and is a hospital-based open-ended prospective cohort study with the aim of assessing drug safety during pregnancy. Pregnant women who set up the pregnancy health records in the first trimester or who seek drug consultation regardless of gestational age in the member hospitals are recruited. Enrolled pregnant women need to be investigated four times, namely, 6–14 and 24–28 weeks of gestational age, before discharge after hospital delivery, and 28–42 days after birth.

Maternal medication exposure during pregnancy is the focus of the CTBC. For drugs, information on the type, name, and route of medication; start and end time of medication; single dose; frequency of medication; dosage form; manufacturer; and reason for medication is collected. The adverse pregnancy outcomes collected in the study include birth defects, stillbirth, spontaneous abortion, preterm birth, post-term birth, low birth weight, macrosomia, small for gestational age, large for gestational age and low Apgar score. CTBC uses an electronic questionnaire for data collection and a cloud system for data management. Biological samples are collected if informed consents are obtained. Multi-level logistic regression, mixed-effect negative binomial distribution regression and spline function regression are used to explore the effect of drugs on the occurrence of birth defects.

**Discussion:**

The findings of the study will assist in further understanding the risk of birth defects and other adverse pregnancy outcomes associated with maternal drug exposure and developing the optimal treatment plans and drug counselling for pregnant women.

**Trial registration:**

This study was approved by the Research Ethics Committee of the West China Second Hospital of Sichuan University and registered at the Chinese Clinical Trial Registry (http://www.chictr.org.cn/index.aspx, registration number ChiCTR1900022569).

**Supplementary Information:**

The online version contains supplementary material available at 10.1186/s12884-021-04073-0.

## Background

Since the thalidomide disaster occurred in the 1960 s, people have paid increasing attention to the medication safety during pregnancy [1, 2]. Drug exposure during pregnancy may cause adverse pregnancy outcomes, including birth defects, spontaneous abortion, foetal growth restriction, stillbirth, premature birth and low birth weight [3–5]. It is estimated that 2–3 % of birth defects are due to drug exposure during pregnancy [6]. Previous studies showed that most pregnant women take at least one drug during pregnancy [7–11], and the proportion of pregnancies involving medication varied from 27 to 93 % in developed countries [12]. The phenomenon is more common than before because of the higher proportion of pregnancies complicated with chronic diseases and inadvertent drug use after unintended pregnancies [3, 13–16]. From 1976 to 2008 in the United States, the frequency of prescription drug use among pregnant women increased by more than 60 % [17]. In 2015, the alphabet category system (ABCDX) established by US Food and Drug Administration (FDA) was discontinued due to many faults [18], and was replaced by a new system called the Pregnancy and Lactation Labelling Rule (PLLR) [19, 20] which emphasizes the importance of assessing drug safety during pregnancy on the basis of evidence [21].

Although some drugs are known to potentially be harmful to foetuses, this issue is far from resolved [22]. The limited knowledge regarding the safety of drugs during pregnancy poses significant challenges in the development of optimal treatment plans and drug counselling for pregnant women [23–27], which even caused excessive terminations of pregnancy [28–30]. The harm caused by drugs during pregnancy to the foetus could be largely preventable if sufficient evidence was available from humans to better understand the risks of medications [31–34].

The types of research commonly used in drug safety studies during pregnancy include case reports, case-control studies, retrospective cohort studies or existing medical databases. Each study design has advantages and disadvantages [3, 11, 35–40]. In contrast, prospective cohort studies can yield complete and reliable information on the safety of drug exposure during pregnancy after marketing. Therefore, the National Center for Birth Defect Monitoring of China (NCBDMC) is establishing a prospective and multicentre cohort called the China Teratology Birth Cohort (CTBC) to bridge the knowledge gap on drug safety during pregnancy.

## Methods and design

The CTBC aims to assess the risk of birth defects and other adverse pregnancy outcomes (stillbirth, spontaneous abortion, preterm birth, post-term birth, low birth weight, macrosomia, small for gestational age, large for gestational age, low Apgar score) associated with maternal drug exposure. It was launched in January 2019 and is a hospital-based open-ended prospective cohort study designed to evaluate drug safety during pregnancy, and it is expected to recruit at least 300,000 participants. Pregnant women who meet the inclusion and exclusion criteria (see the following section) are enrolled in the first trimester (usually between 6 and 14 weeks gestational age) of pregnancy when they first receive antenatal care in the member hospitals, and each enrolled pregnant woman is followed up three times, namely, in the second trimester (usually between 24 and 28 weeks gestational age), before discharge from a member hospital after delivery and within 42 days after delivery (usually between 28 and 42 days after delivery). Enrolled women complete four investigations in total under face-to-face instruction from a trained investigator. Biological samples, such as maternal peripheral blood, maternal urine, placenta, umbilical cord and diseased tissue, are collected if informed consent is obtained from the pregnant woman.

### The member hospital and the project executive team

**Fig. 1:**
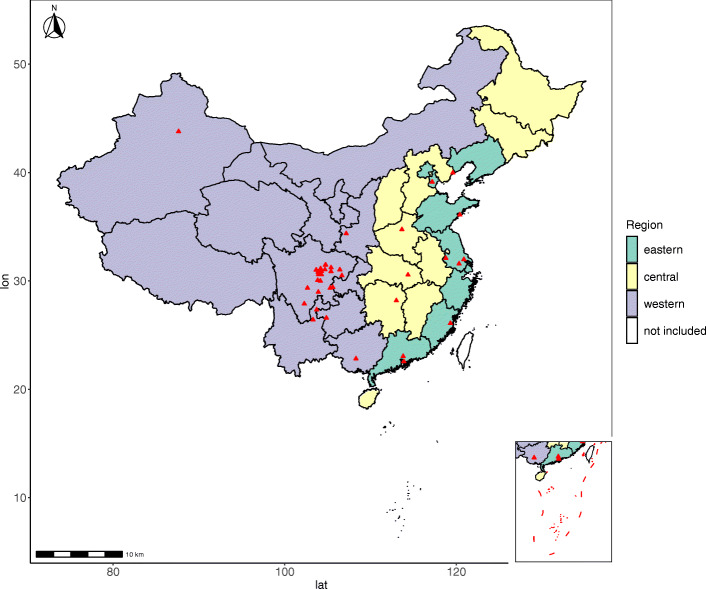
Map of member hospitals in the China Teratology Birth Cohort 2019–2021. Map source: Resource and Environment Science and Data Center

Hospitals can freely apply to participate in the project as long as they meet the following conditions: (1) it must be a county-level or higher hospital with the departments of obstetrics and paediatrics, and (2) it must be a member hospital of the national or provincial birth defect monitoring system in China [41]. As of June 30, 2021, a total of 47 hospitals (including 17 county-level hospitals, 23 prefecture-level hospitals, and 7 provincial-level hospitals) located in 21 provinces in China have participated in the study (Fig. 1).

An executive team of 5–6 staff members with a medical background is set up in each member hospital. The team consists of one manager, at least three investigators and one quality inspector. The manager is responsible for coordinating the obstetrics, pharmacy, paediatrics and other departments involved in the study, as well as organizing the investigators and quality controllers to implement the project as required. The investigators are responsible for recruiting and informing the patients, urging the enrolled pregnant women to come to the member hospital for regular prenatal examinations, directing them to complete the questionnaire and collecting information on the pregnancy outcomes. The quality inspectors are responsible for checking the data quality and data management.

### Study population and recruitment methods

The CTBC has developed clear and reasonable inclusion and exclusion criteria for the research subjects, without regard to occupation, education, and any other social demographic characteristics. Two types of pregnant women are recruited in the study: (1) pregnant women who are establishing their pregnancy health record during the first trimester at the pregnancy health record room of a member hospital and also plan to undergo prenatal care and deliver in the same member hospital; and (2) pregnant women who undergone a drug consultation and has established or plan to establish pregnancy health record of a member hospital and also plan to undergo prenatal care and deliver in the same member hospital. Electronic informed consent is obtained before a subject is enrolled in the study. However, women who are under 18 or cannot complete the investigation due to mental problems or disabilities and those who do not wish to participate will not be recruited in the study. It should be emphasized that pregnant women without drug exposure before the first antenatal visit can also be enrolled.

Pregnant women are recruited in several ways in the study. Eligible pregnant women are recruited by investigators, obstetric doctors and drug consultants. Furthermore, posters are placed in member hospitals to assist in the enrolment of pregnant women.

### Data collection

#### Investigation contents

A structured questionnaire has been designed to collect detailed information for each enrolled pregnant woman. It consists of four sections (see Table 1). Section A investigates the demographics of pregnant women and basic information about the pregnancy. Section B investigates the pre-pregnancy living habits, diseases and drug exposure information of the pregnant women and their husbands. Section C investigates information on maternal complications and drug exposure of the pregnant women. Section D investigates pregnancy outcomes and infant health status within 42 days after birth.

**Table 1:** The questionnaire content of the CTBC

Section	Overview	Content	Pregnancy period
Section A: basic information on pregnant women and pregnancy	Collects demographic information on pregnant women, basic information on this pregnancy, pregnancy history, abnormal fertility history, and family history of congenital malformation and genetic disease.	Includes the patient’s name, nationality, ID number, date of birth, telephone number, address, educational level, family income, date of her last menstrual period, length of her menstrual cycle, method of conception, date of ultrasound examination in early pregnancy, gestational age, gravidity, parity, history of miscarriage, history of birth defects, history of stillbirth, family history of heredity, and height and weight before pregnancy.	First-trimester pregnancy
Section B: living habits, disease and drug exposure information on pregnant women and their husbands before pregnancy	1. Collects data on smoking, drinking, and folic acid supplementation from 3 months before pregnancy to the first 3 months after pregnancy. 2. Collects information on existing disease and exposure to drugs in pregnant women and their husbands within 6 months before conception.	1. Includes smoking, drinking and types, times, days and frequency of supplemental folic acid. 2. Includes the name of the disease, date of onset, current status of the disease (cured, improved, uncured, aggravated), name of the drug, method of administration, start and end time, days on medication, single dose, frequency, dosage form, manufacturer, and reason for use.	First-trimester pregnancy
Section C: diseases and drug exposure information during pregnancy	Collects information on illnesses and medications used by pregnant women and their husbands during pregnancy.	Includes the name of the disease, date of onset, current status of the disease (cured, improved, uncured, aggravated), name of the drug, method of administration, start and end time, days on medication, dosage, frequency, dosage form, manufacturer, and reason for use.	First- and second-pregnancy trimester and discharge before hospital delivery
Section D: pregnancy outcomes and condition of infants within 42 days after birth	Collects data on pregnancy outcomes and conditions of the infants within 42 days after birth.	Includes the date, place and manner of termination of pregnancy, cause of miscarriage or induction, number of births, foetal status (sex, birth status, weight, body length, head circumference, Apgar score), birth defects (name, location, size, diagnostic basis, diagnostic time and diagnostic hospital), and newborn status, hospitalization and birth defects within 42 days of birth.	discharge before hospital delivery or within 28–42 days after delivery

Of all the above information, the detailed information on maternal complications, medication exposure and pregnancy outcomes are the most important. Regarding maternal complications, the CTBC collects the disease diagnosis, time of onset, and current disease status (cured, improved, uncured, aggravated). Regarding medication, the CTBC collects the type (traditional Chinese medicine, Western medicine), name, route of medication, start and end time of medication, single dose, frequency of medication, dosage form, manufacturer and reason for taking the medication.

#### Investigation tool

The popularization of smart phones in the Chinese population has made electronic data collection an alternative data collection method in China. The study mainly relies on electronic questionnaires for data collection, which is based on a popular social app in China called WeChat. Compared with traditional paper-based questionnaires, electronic questionnaires are more efficient in data management and can avoid more logical errors [42–44]. Under special conditions, paper-based questionnaires are allowed, and the data need to enter into the cloud system later. An ID number is set for the unique identification code of each enrolled woman.

#### Investigation timing

**Fig. 2:**
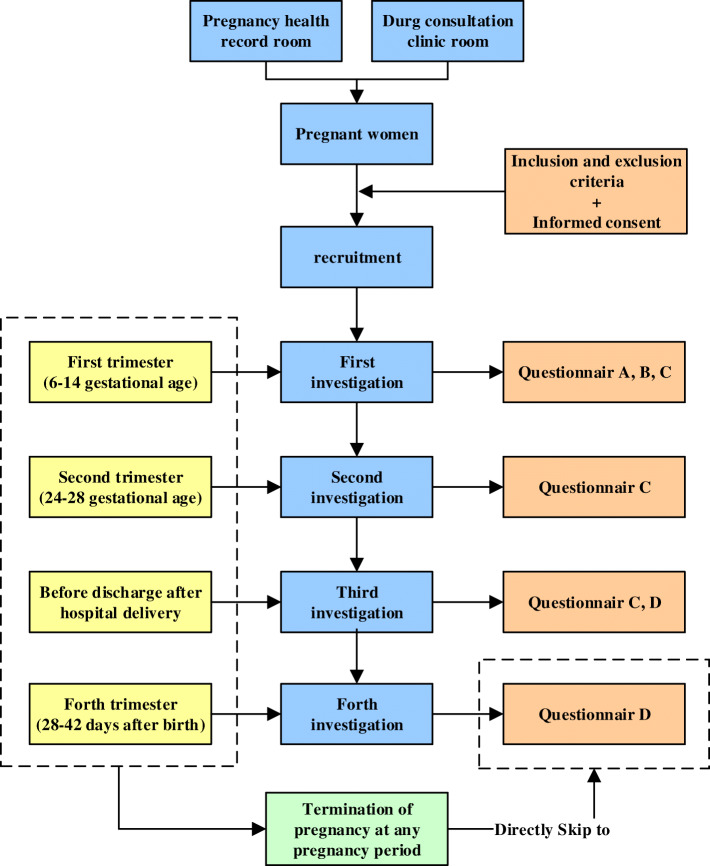
Study design and investigation timing of the CTBC

Each pregnant woman enrolled in the pregnancy health room needs to complete four investigations under the guidance of the investigator (Fig. 2). The first investigation is completed when the pregnant woman comes to the member hospital to establish the pregnancy health record in the first trimester (usually between 6 and 14 weeks gestational age). Enrolled pregnant women need to complete sections A, B, and C of the questionnaire at the first investigation. The second investigation is conducted during the second trimester (usually from 24 to 28 weeks gestational age) when the vast majority of pregnant women come to the hospital for glucose tolerance screening, and the enrolled woman need to repeatedly complete only questionnaire C. Before the enrolled women are discharged from the member hospitals after delivery, the third investigation needs to be completed, which consists of completing both sections C and D of the questionnaire. Within 28–42 days after delivery, the last investigation is carried out when the child of an enrolled woman undergoes a routine physical examination. Every pregnant woman enrolled in the drug consultation clinic room needs to be surveyed by a drug consultant. The four surveys are carried out in the drug consultation clinic room, and the investigation timing is the same as described above.

In the case of spontaneous abortion, artificial abortion or induced labour, pregnant women can skip the middle sections of the questionnaire and complete section D (pregnancy outcome) directly. If the enrolled pregnant women do not go to the member hospital on time to complete the next investigation or do not give birth in the member hospital, investigators will try to conduct the investigation by phone. If an enrolled pregnant woman refuses to answer the phone or refuses to respond to a telephone survey on multiple occasions, she will be deemed as lost to follow-up. For those who are lost to follow-up, the CTBC can obtain their information regarding disease and drug exposure through the medical records in the member hospitals.

During the first survey in the first trimester and the second survey in the second trimester, maternal peripheral blood and urine are collected from the enrolled women after obtaining informed consent. The placenta, umbilical cord and diseased tissue of recently delivered newborns who are diagnosed with a major birth defect are collected. To obtain a 1:1 proportion of cases and normal foetuses, the CTBC also collects the placenta, umbilical cord of normal foetuses whose parents sign the informed consent.

### Drug exposure measurement

It is important to identify the drug name, exposure time and cumulative exposure dosage. In China, the types of medicines can be divided into Western medicine, Chinese medicine, Chinese patent medicine and health products. Among them, the types of medicines we focus on are Western medicine and Chinese patent medicine. For Chinese medicine, we investigate only whether pregnant women have taken Chinese medicine rather than the specific ingredients included in the Chinese medicines they have taken. For health products, although we have to carry out related investigations, they are not our focus.

The CTBC considers any medication taken during any pregnancy period as a drug exposure, even if it is taken only once. Identification of the time of drug exposure is based on the accurate confirmation of gestational age of the enrolled women. The study collects the time of the last menstrual period of pregnant women, the examination time of prenatal ultrasound and the foetal gestational age confirmed by prenatal ultrasound in the first trimester. Therefore, the gestational age inferred by the time of the last menstrual period and ultrasound examination, combined with the date of drug exposure, determines the pregnancy period of drug exposure. The cumulative exposure dose can be calculated from the single exposure dose, exposure frequency, exposure start and end time collected by us.

To ameliorate the problem of inaccurate information recall regarding diseases and drugs, the following methods are used in the study. First, the common diseases and drug names are set with drop-down options in the electronic questionnaire, which could also reduce input errors. Second, each member hospital prints out photographs of names and boxes of common drugs locally to help enrolled women recall details. Third, the pregnant women are advised to recall the start and end time of illness and medication use through important festivals or iconic events. Fourth, if the above methods still fail to allow the pregnant woman to recall the drug information, the pregnant women are asked to go home and look for the medicine box or medical record. In addition, if the drugs not in the drop-down menu are exposed, pregnant women can enter the drug name and other information into the electronic questionnaire, and we will code them.

Another difficulty in the investigation of exposure measurement is how to fill in the time of drug exposure across different pregnancy periods. The CTBC requires that the end time of drug use in each survey be filled up to the day of the survey at most. In the next survey, the earliest time when the drug started to be used is filled in the first day after the last survey. In addition, we have set the above filling rules in the electronic questionnaire. In this manner, the time of the two surveys can be linked, and drug exposure of the pregnant women enrolled in the study can be observed throughout their pregnancy. Pregnant women with chronic diseases usually go through the process of taking, stopping, and returning to their medications during pregnancy. This rule also prevents pregnant women with chronic diseases from filling in the end time of drug exposure directly according to their due date of birth in the first survey.

### Pregnancy outcome measurement

Accurate measurement of gestational age at delivery, birth weight, birth defects and related adverse pregnancy outcomes is a key component of this study. The determination of the gestational age at delivery is the same as above and depends on the last menstrual time of the enrolled women, the examination time of prenatal ultrasound and the foetal gestational age as confirmed by prenatal ultrasound in the first trimester as above. The birth weight of newborns is uniformly measured with an electronic scale, and the measurement is accurate to 10 g.

The CTBC is concerned with almost all adverse pregnancy outcomes, such as birth defects, stillbirth, spontaneous abortion, preterm birth, post-term birth, low birth weight, macrosomia, small for gestational age, large for gestational age, and low Apgar score. Regarding adverse pregnancy outcomes, we focus most on birth defects. In the study, all birth defects (including major structural abnormalities, minor congenital abnormalities, and functional abnormalities) from the first trimester to 42 days after birth need to be identified, regardless of whether a pregnancy ends in live birth, foetal death, or spontaneous abortion or whether an elective termination is performed. Prenatal ultrasound screening and prenatal diagnosis are the main means to detect birth defects before birth, but the diagnosis of birth defects needs to be confirmed by clinical observation and physical examination of a newborn. Clinical observation of a newborn includes the observation of its spirit, complexion, limb movement, breathing, diet, urine and faeces. Neonatal physical examination should first include a body surface examination, examination of the body surface without deformity, and examination of each part of the organ individually. Physical examination can also yield some information on obvious visceral malformations, and the diagnosis of some visceral malformations should be combined with laboratory examination and clinical symptoms. For infants who are discovered and suspected of having a birth defect within 42 days after birth but are not yet diagnosed, the follow-up will continue until a diagnosis is made. If certain birth defects cannot be clearly diagnosed in member hospitals, a specialist consultation will be conducted to achieve a diagnosis. Regarding the measurement methods of birth defects, we refer to the National Birth Defects Prevention Network (NBDPN) in America [45] and China Maternal and Child Health Monitoring Manual [46]. For definitions of other adverse pregnancy outcomes, please see Additional file 1.

### The data standardization

#### Drug standardization

The Anatomical Therapeutic Chemical (ATC) classification, a system developed by the World Health Organization (WHO) Collaborating Centre for Drug Statistics Methodology, is recommended for drug coding worldwide [47]. Based on ATC drug code classification standards, the Ministry of Human Resources and Social Security of China formulated the “Social Insurance Drug Classification and Code” industry standard [48], which includes the code of Western medicine and Chinese patent medicine. The “Social Insurance Drug Classification and Code” industry standard is used as reference for drugs standardization.

#### Disease standardization

The study uses the tenth revision of the International Classification of Diseases (ICD-10) to standardize and record maternal diseases and related diseases occurring at the time of pregnancy termination (e.g., birth defects, premature labour, miscarriage, stillbirth, macrosomia) [49]. We have performed additional coding on the basis of ICD-10 to investigate cases that have only relevant symptoms or abnormal laboratory test indicators but have not been diagnosed with a disease, such as low luteal function, low progesterone hormone, high uric acid, high prolactin, and high or low thyroid hormone.

The study also adds the drug and disease codes to the electronic questionnaire and cloud system such that the names and codes of the collected drugs and diseases are directly standardized.

### Data management and cloud system

**Fig. 3:**
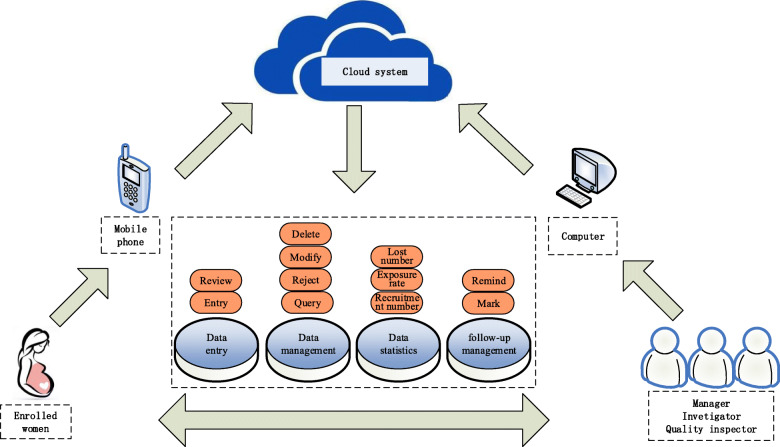
Cloud system operation diagram

We have designed a cloud system related to the electronic questionnaire, which plays an important role in data management (Fig. 3). The cloud system has for functional modules of data entry, data management, statistics and follow-up management. The questionnaire has been embedded in the cloud system, and it can be used by both pregnant women and investigators for data entry. The module of data management can perform the essential functions of data query, rejection, modification and deletion. The system also provides a data statistics function, which can automatically count the number of enrolments, drug exposure rate, disease exposure rate and the number of patients lost to follow-up to facilitate dynamic tracking of the project implementation progress. Moreover, the cloud system can remind investigators which pregnant women should undergo a subsequent investigation.

### Quality control measures

Strict quality control measures are taken before, during and after the implementation of the project as following:

1. The NCBDMC provides standardized training for each member hospital project executive team. All member hospitals use standardized survey tools, survey methods, survey techniques and quality control methods.
2. Pregnant women who will participate in regular prenatal care and delivery in member hospitals are selected as the enrolled population. These populations are highly compliant and have a high retention rate.
3. To increase the number of enrolled women and the follow-up rate, gifts are provided to enrolled pregnant women to improve their prenatal care compliance.
4. The electronic medical records or paper maternal health manuals of each enrolled pregnant woman are marked so that investigators can identify the enrolled pregnant women at the next investigation sites.
5. Member hospitals notify the enrolled pregnant women of the time and location of the next survey via text messages or phone calls.
6. The CTBC conducts four rounds of quality checks. First, investigators check the completed questionnaire on site focusing on questionnaire integrity and logic error and immediately modify misinformation. Second, quality inspectors check the data quality every week by comparing the information in the medical record system. Third, the NCBDMC checks the data every day focusing on. Fourth, the NCBDMC conducts on-site quality inspections at member hospitals irregulary. In first and third rounds, the questionnaire integrity and logic error are the focus of data inspection.
7. The NCBDMC conducts on-site quality inspections at member hospitals. The specific process is to comparing the disease and medication records of enrolled pregnant women in the hospital medical record system and access all department records in the hospital that may identify infants with congenital anomalies, such as delivery records, neonatology records, prenatal screening and diagnosis records, birth defect monitoring records, newborn screening records, death records and laboratory reports.

### Data analysis

Most importantly, the relationship between maternal drug exposure and adverse pregnancy outcomes will be assessed in this study. It is also possible to explore the relationship between drug exposure and adverse pregnancy outcomes by comparing the drug-taking group with a certain disease and the non-medicating group suffering from the disease. Moreover, the relationship between the period of pregnancy during drug exposure, the duration of drug exposure, the cumulative dose of drug exposure and adverse pregnancy outcomes also warrants investigation. Finally, grouping according to exposure to drug A and drug B can enable a comparison of the relationship between the two drugs and adverse pregnancy outcomes.

In addition, matching, propensity scores and other methods can be used to control the confounding effects between drugs and adverse pregnancy outcomes, and multi-level logistic regression, mixed-effect negative binomial distribution regression and spline function regression can be used to explore the effect of modification and interaction effects between drugs and diseases on the occurrence of adverse pregnancy outcomes.

## Discussion

This is a prospective and multicentre cohort study aiming to bridge the knowledge gap on drug safety during pregnancy, including 47 member hospitals from 21 provinces in China since 2019. In the future, the CTBC will continue to increase the number of member hospitals and the number of enrolled pregnant women and plans to collect data on the severity of maternal disease and long-term development outcomes of children. Based on the CTBC, research on the early warning of teratogenic risks and on standardized services for the prevention and control of clinical teratogenic risks can also be carried out, such as the formulation of risk classification principles of drug use during pregnancy, standardized process of clinical drug information consultation service and guidelines for safe drug use during pregnancy.

The study has several advantages. First, the CTBC is a prospective cohort study, so it has less recall bias and stronger causal arguments and can quantitatively analyse the risk between exposed drug and adverse pregnancy outcomes [50] .49 s, enrolled women fill out the questionnaire by themselves under the guidance of the investigators, to enable the collection of information about prescription drug, over-the-counter medication, and Chinese patent medicine exposure during pregnancy. Currently, there is very little evidence on the safety of Chinese patent medicines during pregnancy. Third, detailed information on exposed drugs and important potential confounding factors is collected, which is helpful for analysing the effects of drugs on adverse pregnancy outcomes. This method is very advantageous compared with many retrospective observational studies and studies conducted using medical databases.

This study has three main limitations. First, following the China Maternal and Child Health Career Development Report in 2019 [51], the prenatal check-up rate in China has reached 96.6 % and the hospital delivery rate has reached over 99 %. The CTBC is designed as a hospital-based birth cohort, which is advantageous with respect to recruitment and follow-up. This cohort is significantly different from a community-based birth cohort. However, because the postpartum visit time for Chinese babies to the hospital is 28–42 days, birth defects found within 42 days of birth can be identified, and congenital abnormalities after 42 days of birth are ignored. Second, infectious diseases, mental diseases and related treatment information are difficult to obtain because pregnant women with these diseases go to designated infectious disease hospitals or psychiatric hospitals for treatment, prenatal care and delivery, and this research has not yet included this type of hospital. However, infectious diseases, mental illnesses and drug exposure are still important research aspects of drug safety during pregnancy. Third, patients with early abortions are under-represented in this birth cohort. Most of the first questionnaire surveys in this study are implemented during gestational weeks 10–14. No special survey is used for pregnant women with an early abortion before 8 weeks, and this group of women is considered a high-risk group for early-term abortion.

In the world, the role of most national pharmacovigilance centres in monitoring drug safety during pregnancy is limited, and the Terminology Information Service (TIS) was established in many countries, such as The European Network of Teratology Information Services (ENTIS) [52] and MotherToBaby (Organization of Teratology Information Specialists, OTIS) [53], and proved to be effective [54, 55]. However, to date, China does not have a similar institution or organization. The CTBC can be used as a powerful supplement to pharmacovigilance centres in the future.

## Supplementary Information

**Additional file 1.**

## Data Availability

Not applicable.

## Abbreviations

CTBC: China Teratology Birth Cohort
FDA: US Food and Drug Administration
PLLR: Pregnancy and Lactation Labelling Rule
NCBDMC: National Center for Birth Defect Monitoring of China
NBDPN: National Birth Defects Prevention Network
ATC: Anatomical Therapeutic Chemical
WHO: World Health Organization
ICD-10: tenth revision of the International Classification of Diseases
TIS: Terminology Information Service
ENTIS: European Network of Teratology Information Services
OTIS: Organization of Teratology Information Specialists
