# Supplementary material for: Protocol of a prospective and multicentre China Teratology Birth Cohort (CTBC): association of maternal drug exposure during pregnancy with adverse pregnancy outcomes
Source: BMC Pregnancy Childbirth. 2021 Sep 1;21:593. doi: 10.1186/s12884-021-04073-0 (PMC8411516; doi:10.1186/s12884-021-04073-0)
Supplement: Supplementary file 1 — Additional file 1. [file 12884_2021_4073_MOESM1_ESM.docx]

**SUPPLEMENTARY MATERIAL**

**Additional table 1**

| **Adverse pregnancy outcomes other than birth defects** | **Definition** |
| --- | --- |
| Stillbirth | Stillbirth is defined as foetal death at 28 weeks of gestation or more, or with a birth weight of 1,000 g or more. |
| Spontaneous abortion | Spontaneous abortion is defined as termination of pregnancy at less than 28 weeks or a foetus weight less than 1000 g. |
| Preterm birth | Preterm birth is defined as a live birth between 22 and 36 weeks of gestation. |
| Postterm birth | Postterm birth is defined as birth that occurs after 42 completed weeks (294 days) of gestation in the study |
| Low birth weight | Low birth weight (LBW) is defined as a birth weight less than 2500 g, and macrosomia is defined as a birth weight more than 4000 g. |
| Macrosomia | Macrosomia is defined as a birth weight beyond 4,000 g, regardless of the gestational age. |
| Small for gestational age | Small for gestational age is defined as a birth weight equal to or less than the 10th percentile for a given gestational age |
| Large for gestational age | Large for gestational age is defined as a birth weight equal to or more than the 90th percentile for a given gestational age |
| Low Apgar score | Low Apgar score is defined as a 1 or 5 minute Apgar score less than 7 points. |
